# Sexually Divergent Mortality and Partial Phenotypic Rescue After Gene Therapy in a Mouse Model of Dravet Syndrome

**DOI:** 10.1089/hum.2019.225

**Published:** 2020-03-17

**Authors:** Yosuke Niibori, Shiron J. Lee, Berge A. Minassian, David R. Hampson

**Affiliations:** ^1^ Department of Pharmaceutical Sciences, Leslie Dan Faculty of Pharmacy, University of Toronto, Toronto, Canada; ^2^ Department of Pediatrics, University of Texas Southwest Medical Center, Dallas, Texas; ^3^ Department of Pharmacology and Toxicology, Faculty of Medicine, University of Toronto, Toronto, Canada.

**Keywords:** adeno-associated virus, autism, cerebral spinal fluid, epilepsy, neurodevelopmental disorder, SCN1A, SCN1B

## Abstract

Dravet syndrome (DS) is a neurodevelopmental genetic disorder caused by mutations in the *SCN1A* gene encoding the α subunit of the NaV1.1 voltage-gated sodium channel that controls neuronal action potential firing. The high density of this mutated channel in GABAergic interneurons results in impaired inhibitory neurotransmission and subsequent excessive activation of excitatory neurons. The syndrome is associated with severe childhood epilepsy, autistic behaviors, and sudden unexpected death in epilepsy. Here, we compared the rescue effects of an adeno-associated viral (AAV) vector coding for the multifunctional β1 sodium channel auxiliary subunit (AAV-NaVβ1) with a control vector lacking a transgene. We hypothesized that overexpression of NaVβ1 would facilitate the function of residual voltage-gated channels and improve the DS phenotype in the *Scn1a*^+/−^ mouse model of DS. AAV-NaVβ1 was injected into the cerebral spinal fluid of neonatal *Scn1a*^+/−^ mice. In untreated control *Scn1a*^+/−^ mice, females showed a higher degree of mortality than males. Compared with *Scn1a*^+/−^ control mice, AAV-NaVβ1-treated *Scn1a*^+/−^ mice displayed increased survival, an outcome that was more pronounced in females than males. In contrast, behavioral analysis revealed that male, but not female, *Scn1a*^+/−^ mice displayed motor hyperactivity, and abnormal performance on tests of fear and anxiety and learning and memory. Male *Scn1a*^+/−^ mice treated with AAV-NaVβ1 showed reduced spontaneous seizures and normalization of motor activity and performance on the elevated plus maze test. These findings demonstrate sex differences in mortality in untreated *Scn1a*^+/−^ mice, an effect that may be related to a lower level of intrinsic inhibitory tone in female mice, and a normalization of aberrant behaviors in males after central nervous system administration of AAV-NaVβ1. The therapeutic efficacy of AAV-NaVβ1 in a mouse model of DS suggests a potential new long-lasting biological therapeutic avenue for the treatment of this catastrophic epilepsy.

## Introduction

Dravet syndrome (DS) is a genetic neurodevelopmental disorder associated with severe spontaneous tonic/clonic seizures, developmental delay, and autism-like behaviors. A particularly morbid feature of the syndrome is that 15–20% of patients succumb to sudden unexpected death in epilepsy (SUDEP), through which the patient expires after a seizure.^1^ SUDEP is thought to be caused by seizure-induced respiratory and cardiac failure.^2–4^ The incidence of this genetic disorder has been reported to range from 1 in 15,000 to 1 in 45,000 (Refs.^5,6^). It is most severe during childhood, with the epileptic seizures waning somewhat as the patient ages,^7^ while the autistic-like symptoms persist lifelong.^8^ The syndrome is generally resistant to antiepileptic drugs, making effective treatment difficult.^9^ Antiseizure medications such as stiripentol and cannabidiol have conferred some improvements in seizure control, however, no medication alters the severe developmental morbidity of the disease.

Approximately 80% DS patients possess a mutation in the *SCN1A* gene. The mutations are dispersed throughout the coding region of one allele, thereby causing haploinsufficiency of the encoded protein, the sodium channel NaV1.1 α subunit.^10,11^ NaV1.1 is widely distributed throughout the central nervous system (CNS) at low-to-moderate density.^12,13^ Mice with a heterozygous deletion of *Scn1a*, a widely used animal model of DS, display heat-induced seizures, spontaneous tonic/clonic seizures, autism-like behaviors, and SUDEP.^11,14^ Notably, conditional *Scn1a*^+/−^ mice that have reduced Nav1.1 protein only in GABAergic interneurons show an overall phenotype similar to global *Scn1a*^+/−^ mice.^13,15,16^ These observations and others indicate that the decrease in sodium channel activity in GABAergic interneurons of *Scn1a*^+/−^ mice underlies the DS phenotype.

In light of the shortcomings of small-molecule-based pharmacotherapy, we sought to develop a potential treatment for DS using viral vector-mediated gene therapy based on expression of recombinant sodium channel proteins. For CNS disorders, the preferred type of vector for both preclinical and clinical research is the recombinant adeno-associated viral (AAV) vector.^17–19^ However, a limitation of AAV-based vectors is the size restriction of the inserted foreign DNA; 4.7 kb for single-stranded vectors. Because the coding region of the NaV1.1 α subunit is too large (∼6 kb) to incorporate into a recombinant AAV vector, we explored an alternative approach entailing the overexpression of a smaller sodium channel auxiliary subunit. Voltage-gated sodium channels, such as NaV1.1, are heterotrimeric protein complexes consisting of a single large pore-forming α subunit in combination with two much smaller β subunits.^20^ The noncovalently linked β1 subunit has been shown to modulate the function of the α subunits by enhancing ion flow through α subunits, and by promoting the trafficking of the α subunits from an intracellular pool to the cell surface.^21^

We hypothesized that overexpression of NaVβ1 would facilitate the activity of residual sodium channel subunits in *Scn1a*^+/−^ mice and mitigate the DS phenotype. An AAV vector encompassing a promoter based on a GABAergic neuron expressing gene, *Gad-1*, and the coding region of mouse β1 (AAV-NaVβ1), was injected into the neonatal mouse brain. Seizure testing was conducted in juvenile mice, while behavioral testing was carried out on adult mice. Our findings reveal sex differences in DS susceptibility to SUDEP, and are the first to demonstrate reduced mortality and partial correction of abnormal behaviors in a mouse model of the disorder using viral vector-mediated gene therapy.

## Experimental Procedures

### Mice and genotyping

*Scn1a* heterozygous (129.Scn1a^+/−^) mice on the 129S6/SvEvTac genetic background (129S Scn1a^tm1Kea^/Mmja^22^; Jackson Laboratory) were crossed with C57BL/6J mice (Jackson Laboratory) to generate 129 × B6 F1 hybrid offspring. Half of the breeder pairs consisted of wild-type (WT) female C57BL/6J mice crossed with male mutant mice, and the other half consisted of the converse, female SV 129 mutant mice crossed with male WT C57BL/6J mice. These 129 × B6 *Scn1a*^+/−^ F1 hybrid mice and WT littermates were used in all experiments. No incidences of SUDEP were observed in 129.*Scn1a*^+/−^ mice. All mice were housed in a 07:00–19:00 light/dark cycle with *ad libitum* access to food and water, and all procedures were approved by the University of Toronto Animal Care Committee and were carried out in compliance with the Canadian Council on Animal Care guidelines. Genomic DNA was isolated from tail clips and the *Scn1a* genotype was determined by PCR. DNA extraction and PCR amplification were performed using the MyTaq Extract-PCR Kit (BIOLINE). Fifteen microliters of the PCR mixture (1 μL of genomic DNA, 1.5 μL each of 10 μM primers, 7.5 μL of 2xMyTaq HS Red Mix, and 2 μL of distilled water) was used for PCR, and a four-step cycle PCR protocol (first: 95°C for 3 min, second: 95°C for 15 s, third: 58°C for 15 s, fourth: 72°C for 20 s; repeat second to fourth steps 30 times) was used for PCR amplification. The primers for WT and Scn1a targeted alleles were (1) common forward primer; 5′-AGTCTGTACCAGGCAGAACTTG-3′, (2) WT-specific reverse primer; 5′-CCCTGAGATGTGGGTGAATAG-3′, and (3) *Scn1a* targeted allele-specific reverse primer; 5′-AGACTGCCTTGGGAAAAGCG-3′. These primers amplify a 280 bp product from the WT allele and a 150 bp product from the targeted allele.

### AAV vector generation and virus production

The pAAV9-pGad1-NaVβ1-myc (AAV-Navβ1) vector consisted of a truncated mouse *Gad1* promoter, mouse NaVβ1 gene-fused myc tag at C terminus, a woodchuck hepatitis virus posttranslational regulatory element (WPRE), and rabbit β-globin polyadenylation region. The mouse Gad1 promoter was amplified by PCR using the 5′ primer (5′-gatcGAATTCaCGAGACGCTTCACCCTACAA-3′) and 3′ primer (5′-gcatGGATCCaAGATCTGTCCGGGTGATCCGGTATTT-3′). The mouse Scn1b gene encoding NaVβ1 protein was amplified from an Scn1b encoding DNA plasmid (Origene) by PCR using Top Taq DNA polymerase (Qiagen). Those DNA fragments were flanked by inverted terminal repeats (ITRs) in an AAV-Navβ1 vector. AAV-NaVβ1 vector was amplified and purified by Endotoxin-free Maxi-prep (Qiagen) for AAV production. The AAV empty vector (AAV-EV) was ligated to a cytomegalovirus promoter, WPRE, and rabbit beta-globin poly(A) region flanked by ITRs to insert into an AAV genome. High-titer viruses for AAV-NaVβ1 and AAV-EV were produced at the Vector Core Facility at the University of Pennsylvania.

### AAV injections

On postnatal day (PND) 2, *Scn1a*^+/−^ and WT littermates were injected with either AAV-EV or AAV-NaVβ1 (8 × 10^10^ genome copies/mouse) via bilateral intracerebroventricular (i.c.v.) and intracisterna magna (i.c.m.) routes using custom-made injection needles. The sites of the needle insertion were 1 mm from the mouse midline and 1 mm from lambda for i.c.v. injection, and 2 mm from lambda on the cerebellum along the midline for i.c.m. injection (Supplementary Fig. S1). The cut edge of the needle was directed rostrally for i.c.v. injection and toward the ventral side for i.c.m. injection. The volume of the AAV vector was 1 μL/side for i.c.v injection, and 3 μL for i.c.m. injection administered at a flow rate of 1 μL/min. The needle was left in place for an additional 1 min after the infusion.

### Detection of sodium channel subunits by Western blots

Four- to 6-month-old adult mouse brains were homogenized with a solubilization buffer (10 mM Tris-HCl [pH 6.8], 1 mM EDTA, pH 8.0, 150 mM sodium chloride, 0.2% sodium dodecyl sulfate, 0.5 mM sodium deoxycholate, 1% Triton X-100, and 1 × protease inhibiter in distilled water) using a Heidolph homogenizer. The homogenates were incubated on ice for 1 h and centrifuged at 16,000 *g* for 5 min at 4°C. The protein concentrations were quantified using the Pierce BCA Protein Assay Kit. The supernatants were mixed with lithium dodecyl sulfate (LDS) sample buffer [2 M urea, 1 × LDS buffer (Invitrogen), and 0.1 M dithiothreitol in distilled water]. The samples were incubated for 30 min at 37°C and then stored at −80°C. The solubilized membrane (150 μg for NaV1.1, 30 μg for NaVβ1 and NaVβ1-myc, and 10 μg for GAPDH) fraction samples were resolved on 5% polyacrylamide gels (for NaV1.1), or on 12% polyacrylamide gels (for NaVβ1-myc and NaVβ1 proteins), transferred to nitrocellulose membranes, and incubated in 5% skim milk in wash buffer (1 mM Tris, 150 mM sodium chloride, and 0.2% Tween 20 in distilled water) for 1 h, and then incubated with the following primary antibodies: mouse anti-NaV1.1 (1:1,000, 75-023; NeuroMab) in 0.5% skim milk in wash buffer, rabbit anti-NaVβ1 (1:3,000, D4Z2N; Cell Signaling), or rabbit anti-myc tag (1:3,000, ab9106; Abcam), or rabbit anti-GAPDH (1:10,000, ab9485; Abcam) in 5% skim milk in wash buffer at 4°C overnight. The membranes were incubated with anti-mouse immunoglobulin G (IgG) horseradish peroxidase (HRP) conjugated (1:3,000; Jackson Immunolab), or anti rabbit IgG HRP conjugated (1:3,000; Jackson Immunolab), washed, and incubated with the SuperSignal West Pico PLUS Chemiluminescence Substrate for NaVβ1, myc tag, and GAPDH (Thermo Scientific) and with the SuperSignal West Femto Chemiluminescence Substrate for NaV1.1 (Thermo Scientific). The chemiluminescent signals were captured using an Alpha Innotech Image Analyzer (Alpha Innotech) and the images were quantified by FIJI and ImageJ from NIH.

### Immunocytochemistry

Mice were anesthetized with ketamine/xylazine and perfused with 20 mL of ice-cold 1 × phosphate-buffered saline (PBS) followed by 20 mL of 4% paraformaldehyde solution. The brain was dissected and incubated in the same fixative overnight at 4°C, followed by a 30% sucrose solution in PBS and stored at 4°C. Thirty μm sagittal sections of the brain were obtained and incubated in 1% Triton X-100 solution for 30 min at room temperature. After washing with PBS, the sections were incubated in blocking buffer (3% bovine serum albumin, 5% donkey serum, and 5% goat serum, 0.2% Triton X-100, diluted with 1 × PBS) for 1 h and then incubated overnight at 4°C in the primary antibody. The following primary antibodies were used: rabbit anti-c-myc (1:4,000, ab9106; Abcam), mouse anti-c-myc (1:500, sc-40; Santa Cruz), rabbit anti-GABA (1:1,000, A2052; Sigma), mouse anti-GAD-67 (1:500, MAB5406; Millipore), and rabbit anti-NeuN (1:1,500, ab128886; Abcam) diluted using the blocking solution. The sections were washed 5 × in PBS and incubated with a secondary antibody (1:3,000 anti-rabbit IgG Alexa 594 conjugated or 1:3,000 anti-mouse IgG Alexa 488 conjugated) diluted in blocking solution for 2 h, washed twice, and incubated in 1:2,000 DAPI diluted in PBS, washed in PBS, and mounted on glass slides using ProLong Gold Antifade Mountant (Invitrogen).

Images of the immunostained brain sections were captured by LSM710 (Zeiss equipped an argon diode laser) using 20 × and 40 × objective lenses, and by the BioTek™ Cytation™ 5 Cell Imaging Multi-Mode Reader (BioRad) using 4 × and 20 × lenses. Microscope settings (pinhole, gain, and contrast) were kept constant for all the images in each experiment. Image analysis was carried out using FIJI with ImageJ.

### Mortality analysis

Mortality of *Scn1a*^+/−^ mice and WT littermates with or without AAV injection was monitored daily for 47 days from PND 20 to 56. At PND 21, *Scn1a*^+/−^ mice were weaned into housing cages (30 × 18 × 12 cm) containing two to four mice of the same sex and age per a cage. At least one WT mouse was put with *Scn1a*^+/−^ mice in housing cages. All mice displayed hind limb extensions immediately before death. Statistical analysis was carried out using the log-rank test (Fig. 3A, B), and the log-rank test with Bonferroni's correction (Fig. 3C–E).

### Heat-induced seizures

Test mice were transferred to an experimental room 1 h before the experiment. The experimental box (30 × 18 × 12 cm) was prewarmed at 37°C by a heat lamp from the side. A PND 19 or 20 mouse was placed into the prewarmed experimental box, the video recording commenced, and the temperature was then increased 0.5°C every 30 s by moving the heat lamp toward the box until a tonic seizure was observed, or the box temperature reached 42°C.

### Video monitoring and analysis of spontaneous seizures

An *Scn1a*^+/−^ and a WT mouse littermate (age and sex matched) were placed in a housing cage (30 × 18 × 12 cm) on PND 19. The movement of the two mice in the cages was recorded by a Pi NoIR Camera V2 with a raspberry Pi 3 model B microcomputer (RS Components, Ltd.) from the top of the cage from PND 20 to 30, or until the *Scn1a*^+/−^ mouse died. After the recording, tonic seizures were observed manually under treatment-blinded conditions. The number of tonic seizures was quantified within 24 h before the death, or in the last 24 h of the 30 days of age in survivors. Statistical analysis for both seizure types was carried out using a two-tailed *t*-test.

#### Behavioral analyses

All behavioral assays were performed with *Scn1a*^+/−^ mice and WT littermates with or without AAV injection. The mice were assessed in the open field, elevated plus maze, rotarod test, and passive avoidance tests with a 1-week interval between each test (Supplementary Fig. S2). The open field and passive avoidance tests were performed between 10 a.m. and 1 p.m. For the AAV-treated groups, the experimenter was blind to the treatment group. Between all experiments, the apparatus was swiped twice with 70% ethanol.

### Open field test

Eight-week-old mice were placed in a square-shaped, clear, plexiglass box (40 × 40 × 30 cm) surrounded by white paper. Movement was detected by 16 infrared beams per side and recorded for 20 min; light intensity was set at 20 lux (dim light condition). The total distance for the first 5 min. was quantified using Fusion software connected to the clear plexiglass box with the IR beams (Omnitech Electronics, Inc.).

### Elevated plus maze

The elevated plus maze was performed 1 week after the open field test. The dimension of the arms of the elevated plus maze was 5 × 30 cm (San Diego Instruments). The walls on the closed arms were 15 cm high. The center location of the maze was 5 × 5 cm. The platform of the elevated plus maze was 40 cm above a table (light intensity = 200 lux). The mouse was placed at the center location of the maze and movement was recorded for 5 min. with a CCD camera. The time spent in the open arms of the elevated plus maze was quantified by Viewer software (Biobserve GmbH).

### Passive avoidance test

An apparatus of passive avoidance is a plexiglass box (18 × 36 × 18 cm) with a steel grid floor, and a divider with a gate in the middle. One side of the box was dark, covered by black shades, and the other side of the box was brightly illuminated. On the first day, a mouse was placed in the lighted side for 30 s for acclimation; the mouse accessed the dark side by opening a gate. The latency to crossover from the bright side to the dark side was measured. As soon as the mouse entered the dark side, a foot shock was delivered (0.75 mA, 2 s), and the mouse returned to the home cage. The next day the mouse was re-exposed to the lighted side; 30 s later, the gate was opened and the mouse could access the dark (shock) side. The latency to crossover was measured as an indicator of an associative memory formation of a conditioned dark environment and an unconditioned aversive foot shock.

### Statistics analysis

Data comparing two groups were analyzed with a two-tailed, unpaired *t* test. Data with more than two groups were analyzed by one-way or two-way analysis of variance, followed by Tukey's *post hoc* test. The results are presented as the mean ± standard error of the mean.

Due to the high variance in behavioral analyses, outliers were detected using Equations (1) to (3) according to Tukey's fence.

First quartile Q1 = median of the lower half of the behavioral data set

Third quartile Q3 = median of the upper half of the behavioral data set

(1) $$Interquartilerange\left(IQR\right)=Q3-Q1formula$$

(2) Outlierthreshold1=Q1−1.5×IQRformula

(3) Outlierthreshold2=Q3+1.5×IQRformula

Data points below outlier threshold 1 and above outlier threshold 2 were omitted from the data presented as outliers.

## Results

### Endogenous expression of NaV1.1 and NaVβ1, and NaVβ1 transgene distribution after dual i.c.v. and i.c.m. injections

**Figure 1.**
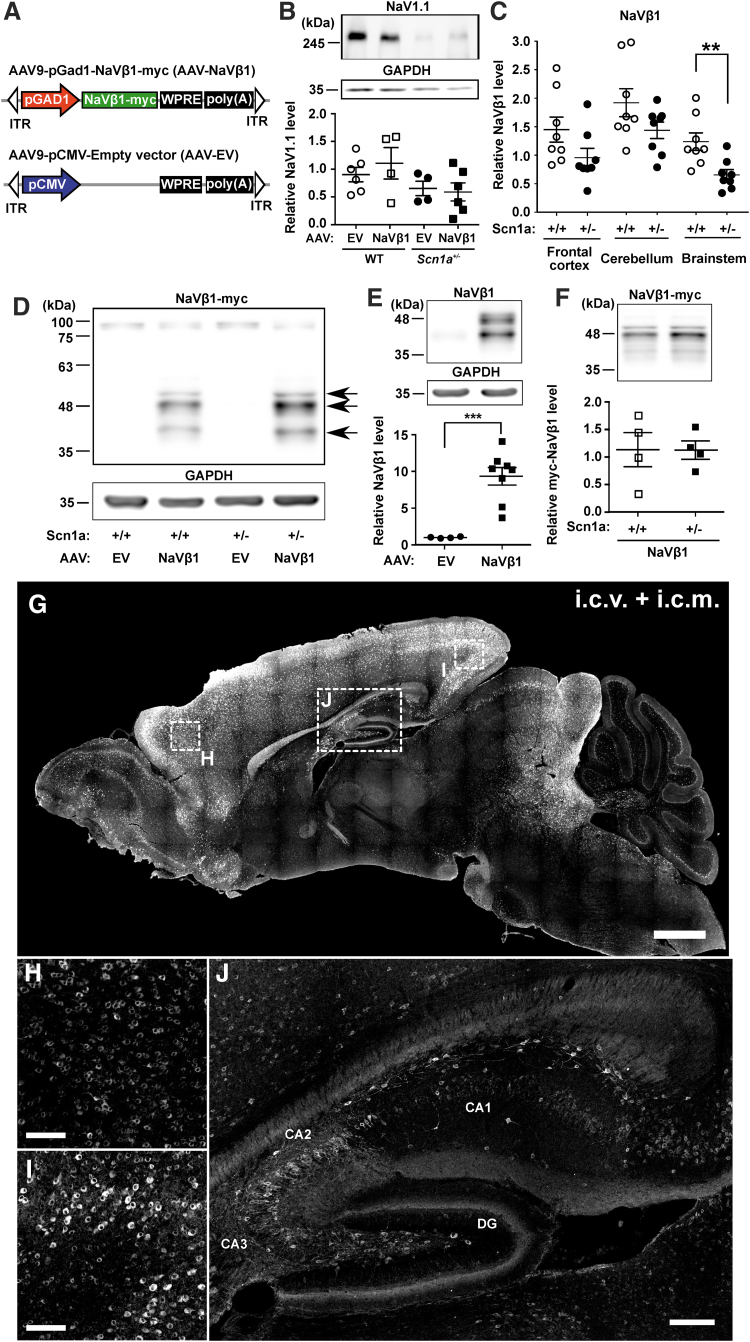
AAV vector design and sodium channel expression in WT and Scn1a^+/−^ mice. **(A)** Schematic diagrams of the two vectors used: AAV-NaVβ1 vector and AAV-EV. **(B)** Endogenous NaV1.1 expression in forebrain of injected WT and Scn1a^+/−^ mice. **(C)** Expression levels of endogenous NaVβ1 as determined by Western blot. **(D)** Western blot of brain sample from mouse injected i.c.v. + i.c.m. with AAV-NaVβ1 and detected with anti-c-myc; *arrow heads* indicate NaVβ1 isoforms. **(E)** Total NaVβ1 (endogenous + transgene) forebrain expression in injected mice. **(F)** Total c-myc-NaVβ1 transgene expression using anti-c-myc in WT versus Scn1a^+/−^ mice. **(G)** Low-magnification sagittal image of NaVβ1 transgene expression in the CNS using anti-c-myc. Higher magnification image of anti-c-myc immunostaining in the frontal cortex **(H)** and visual cortex **(I)**. **(J)** Image of the hippocampus showing c-myc labeling in nonpyramidal cells in CA1, CA2, CA3, and the dentate gyrus. Scale bars, 1 mm for **(G)**, 100 μm for **(H)** and **(I)**, and 200 μm for **(J)**. **p < 0.01; ***p < 0.001. AAV, adeno-associated viral; CNS, central nervous system; EV, empty vector; i.c.m., intracisterna magna; i.c.v., intracerebroventricular; ITR, inverted terminal repeats; WPRE, woodchuck posttranscriptional regulatory element; WT, wild type.

An AAV9 vector encompassing a truncated *Gad-1* gene promoter (coding for Gad-67 protein) and a C-terminal c-myc tagged mouse NaVβ1 coding sequence (AAV-NaVβ1) was generated, along with a control vector missing an expressed transgene (AAV-EV; Fig. 1A). Western blot analyses using an anti-NaV1.1 antibody showed that, as expected, the NaV1.1 protein was reduced in *Scn1a*^+/−^ mice injected with AAV-EV compared with WT mice injected with the same vector; the same pattern in NaV1.1 expression was also observed in WT and *Scn1a*^+/−^ mice injected with AAV-NaVβ1 (Fig. 1B). Unexpectedly, using an anti-NaVβ1 antibody, we found that the expression of endogenous NaVβ1 was reduced in several brain regions in uninjected Scn1a^+/−^ mice compared with WT mice (significant reduction in the brain stem and nonsignificant reductions in the frontal cortex and cerebellum; Fig. 1C). Thus, deletion of one copy of *Scn1a* induced a downregulation of the auxiliary β-1 subunit.

In the AAV treatment groups, mouse pups were treated on PND 2 using a combined i.c.v. + i.c.m. injection of AAV-NaVβ1 (8 × 10^10^ genome vectors per mouse). Western blot analysis using anti-c-myc demonstrated NaVβ1 transgene expression in the mouse brain, through which three protein bands were observed between 43 and 49 kDa, likely corresponding to the unglycosylated, core glycosylated, and fully glycosylated forms of NaVβ^23^ (Fig. 1D). Using the anti-NaVβ1 antibody, the level of total NaVβ1 protein in *Scn1a*^+/−^ mice treated with AAV-NaVβ1 was 8.3 times higher compared with WT mice treated with AAV-EV (Fig. 1E), whereas using an anti-c-myc antibody to assess transgene-generated NaVβ1 revealed similar levels in WT and Scn1a^+/−^ mice treated with AAV-NaVβ1 (Fig. 1F).

Immunocytochemical analysis of the NaVβ1 transgene levels at PND 19 in mice injected with AAV-NaVβ1 on PND 2 showed widespread transduction in the CNS, particularly in the cerebral cortex, hippocampus, olfactory bulb, preoptic area, superior colliculus, and the inferior colliculus, with lower expression in the cerebellum and brain stem (Fig. 1G). Higher magnification images of the frontal (Fig. 1H) and visual cortex (Fig. 1I) revealed robust cellular immunostaining. In the hippocampus, transduced cells included nonpyramidal cells in CA1, CA2, CA3, and nongranule cells in the dentate gyrus (Fig. 1J).

### Cellular specificity of AAV-NaVβ1 transgene expression

Native NaV1.1 α subunit expression is concentrated in GABAergic interneurons.^13,16^ To assess the cellular specificity of the *Gad-1*-based promoter driving NaVβ1-myc transgene expression, we carried out double-label immunocytochemical analysis in the frontal cortex, visual cortex, and hypothalamus, using anti-c-myc to detect NaVβ1-myc, and three cell-type markers. Anti-NeuN was used as a general marker of all neurons, while anti-GABA and anti-Gad-67 were used to label GABAergic neurons. The analysis was conducted in mice injected on PND 2 with AAV-NaVβ1, and brain samples were subsequently collected at two time points; immature mice at PND 17–18 (juvenile to adolescent stage), and adult mice 2–3 months old.

The degree of viral transduction (cell coverage) was examined in the visual cortex, frontal cortex, and hypothalamus. In the immature CNS, cell coverage ranged from 12% in NeuN-labeled cells of the frontal cortex to 57% in Gad-67-labeled cells in the same brain region (Supplementary Fig. S3). In the adult brain, the degree of transduction was lower, ranging from ∼5% to 22% (Supplementary Fig. S3).

**Figure 2.**
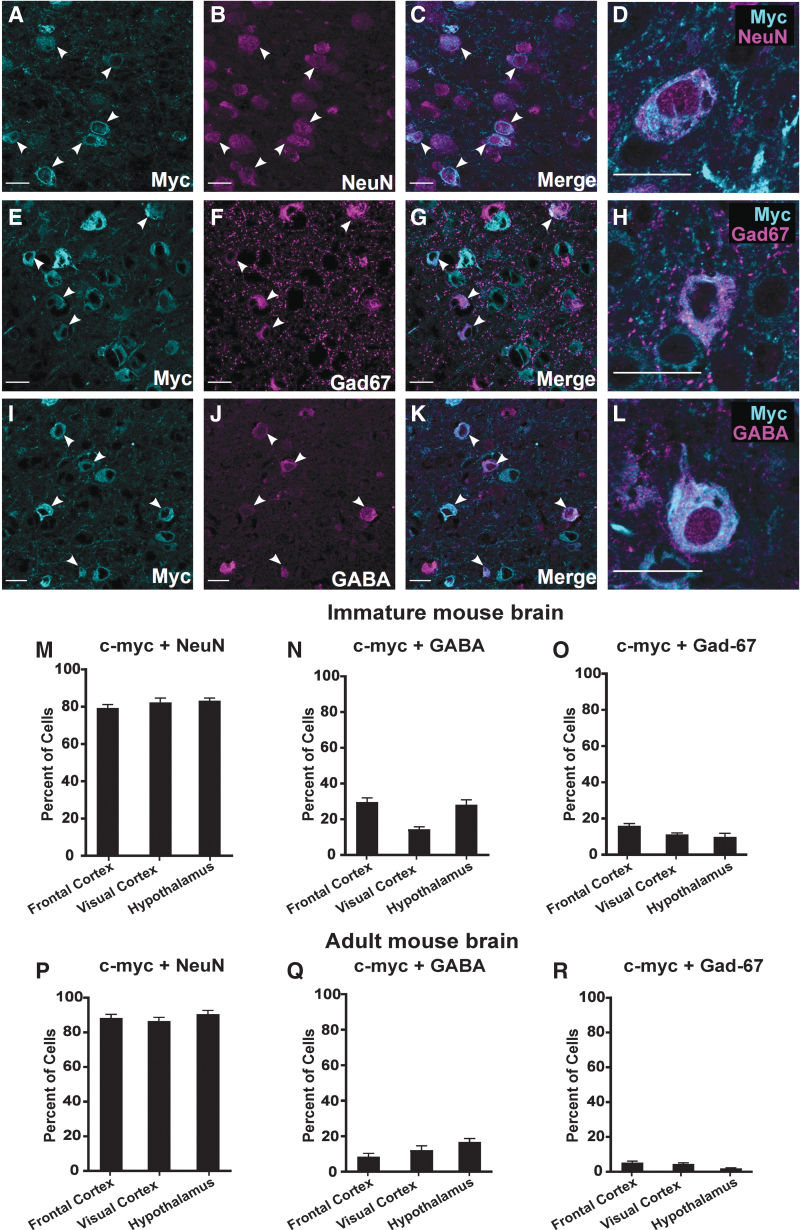
Cellular specificity of NaVβ1 transgene expression. Examples of double-label immunocytochemistry using anti-c-myc and anti-NeuN as a pan-neuronal marker **(A–D)**, anti-Gad-67 **(E–H)**, and anti-GABA for GABAergic interneurons **(I–L)** in the visual cortex. Scale bars = 20 μm in all panels. Summary of quantitative analysis of coexpression of NaVβ1 with the cellular markers NeuN, GABA, and Gad-67 (*arrow heads*) in brain regions of the immature *Scn1a*^+/−^ brain at PND 17–18 **(M–O**; *n* = 4 mouse brains), and in adult brain (**P–R**; *n* = 3 brains). All values are mean ± standard error of the mean. PND, postnatal day.

Double-labeling experiments were also conducted to assess the cell-type specificity of AAV-NaVβ1 transgene expression. In the visual cortex, double-labeling experiments using anti-c-myc for detection of AAV-NaVβ1 and anti-NeuN showed a high level of colocalization (Fig. 2A–D), through which 82.4% and 86.6% of the c-myc-labeled cells were also positive for NeuN in the immature (Fig. 2M) and adult brain (Fig. 2P) samples, respectively. Similar degrees of colabeling with NeuN were seen in the hypothalamus and frontal cortex (Fig. 2M, P). This observation revealed a high level of neuronal selectively of the NaVβ1 transgene. The level of coexpression in Gad-67-positive cells was 11.1% and 4.4%, respectively, in the young and older sample sets (Fig. 2E–H, O, R). The level of coexpression of NaVβ1 in GABA-positive cells in the visual cortex was 14.3% and 12.8% in the immature and mature adult brains, respectively (Fig. 2I–L, N, Q). The degree of colabeling with GABA in the frontal cortex and the hypothalamus in the immature brain was 28–30% (Fig. 2N), and 9–17% in the adult brain (Fig. 2Q). By subtracting the percentage of GAD-67/GABA population from the total NeuN population, we estimated that 50–70% of transgene-labeled cells in the immature brain and 75–85% in the mature brain were non-GABA neurons, most likely pyramidal cells and other glutamatergic neurons. Together, these results indicate high specificity of AAV-NaVβ1 expression in neurons of the immature and mature mouse brain, moderate expression in GABAergic interneurons in the immature CNS, and lower expression in GABA interneurons in the adult CNS.

### Survival of *Scn1a*^+/−^ mice treated with AAV-NaVβ1

**Figure 3.**
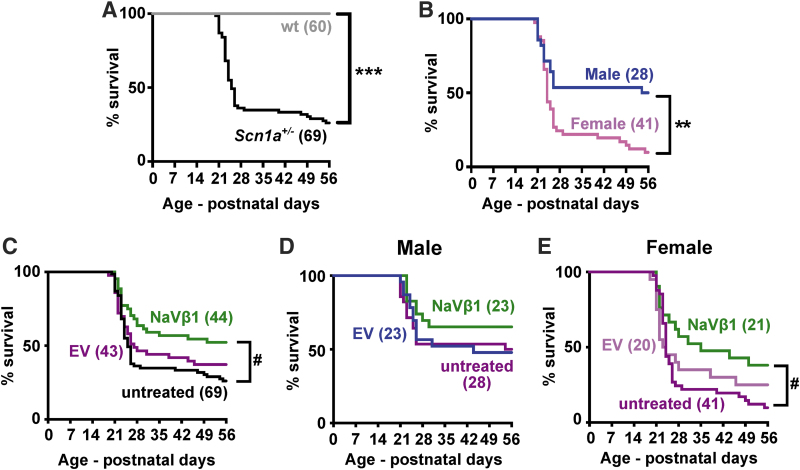
Survival in untreated and treated WT and *Scn1a*^+/−^ mice. The animals were tracked from birth until the beginning of adulthood at PND 56. **(A)** Survival of untreated WT and *Scn1a*^+/−^ mice (males and females combined). Numbers in *parentheses* indicate number of mice in cohort. **(B)** A breakdown of untreated male and female *Scn1a*^+/−^ mice. **(C)** A comparison of untreated *Scn1a* ^+/−^, and *Scn1a*^+/−^ mice treated with AAV-EV or AAV-NaVβ1 (males and females combined). **(D)** Survival in untreated male *Scn1a*^+/−^ versus *Scn1a*^+/−^ mice treated with AAV-EV or AAV-NaVβ1. **(E)** Survival in untreated female *Scn1a*^+/−^ versus *Scn1a*^+/−^ mice treated with AAV-EV or AAV-NaVβ1. **p < 0.01; ***p < 0.001, log-rank test. ^#^*p* < 0.05, log-rank test with Bonferroni's correction. EV: negative control vector.

In untreated *Scn1a*^+/−^ mice, a subpopulation began to die at PND 20 and continued to expire at a high rate until around PND 30, after which the death rate declined (Fig. 3A; χ^2^ = 74.96, *p* < 0.0001). Notably, a comparison of untreated male versus female DS mice demonstrated significantly higher mortality in females compared with males (Fig. 3B; χ^2^ = 10.08, *p* < 0.01). To examine the effects on survival, AAV-NaVβ1-treated DS mice were compared with untreated DS mice and with DS mice injected with a control AAV-EV containing the same AAV9 vector but with no inserted protein coding DNA. Survival of mice injected with AAV-EV was not significantly different from untreated DS mice, while DS mice treated with AAV-NaVβ1 were significantly more likely to survive compared with untreated mice (Fig. 3C; untreated vs. AAV-EV, χ^2^ = 0.93, *p* > 0.05; untreated vs. AAV-NaVβ1, χ^2^ = 8.05, *p* < 0.01; AAV-EV vs. AAV-NaVβ1, χ^2^ = 2.70, *p* > 0.05). A breakdown of the results into males (Fig. 3D; untreated males vs. AAV-EV males, χ^2^ = 0.003, *p* > 0.05; untreated males vs. AAV-NaVβ1 males, χ^2^ = 1.65, *p* > 0.05; AAV-EV males vs. AAV-NaVβ1 males, χ^2^ = 1.42, *p* > 0.05) and females revealed that the prosurvival effect of AAV-NaVβ1 was mainly apparent in the female mice (Fig. 3E; untreated females vs. AAV-EV-treated females χ^2^ = 0.33, *p* > 0.05; untreated females vs. AAV-NaVβ1 females, χ^2^ = 5.88, *p* < 0.05; AAV-EV females vs. AAV-NaVβ1 females, χ^2^ = 1.58, *p* > 0.05).

### The effects of AAV-NaVβ1 on seizure susceptibility

The effects of AAV-NaVβ1 were studied in two seizure tests: spontaneous seizures and heat-induced seizures. To assess the effect of AAV-NaVβ1 on spontaneous seizures, an *Scn1a*^+/−^ mouse was paired with a WT littermate and video recorded on PND 19 until death or PND 30. If the mouse died during the recording, the number of tonic seizures that occurred within 24 h immediately before death was quantified.

**Figure 4.**
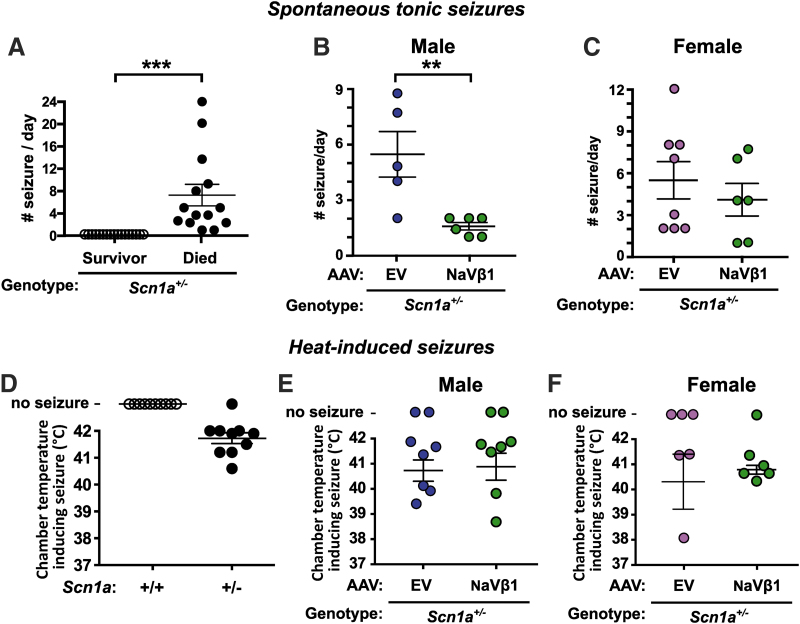
Analysis of spontaneous and heat-induced seizures. **(A–C)** Results from video recordings of mice quantitating spontaneous seizures in untreated *Scn1a*^+/−^ mice **(A)**, in male *Scn1a*^+/−^ mice treated with AAV-EV or AAV-NaVβ1 **(B)**, and in female *Scn1a*^+/−^ versus *Scn1a*^+/−^ mice treated with AAV-EV or AAV-NaVβ1 **(C)**. Analysis of heat-induced seizures in untreated *Scn1a*^+/−^ mice **(D)**, in male *Scn1a*^+/−^ mice treated with AAV-EV or AAV-NaVβ1 **(E)**, and in female *Scn1a*^+/−^ mice treated with AAV-EV or AAV-NaVβ1 **(F)**. **p < 0.01; ***p < 0.001, two-tailed *t*-test.

The seizure frequency within the 24 h before death in untreated *Scn1a*^+/−^ mice ranged from 1 to 24 seizures per day in the mice that died, whereas surviving mice did not display seizures (Fig. 4A; *t* = 4.17, *p* < 0.001, males and females combined). A breakdown based on sex showed that males had an average of 7.8 seizures per day, while females had 6.9 per day (Supplementary Table S1). All of the surviving *Scn1a*^+/−^ mice, both untreated and injected with AAVs, did not display tonic/clonic seizures at PND 30 (Supplementary Table S1). Male *Scn1a*^+/−^ mice injected with AAV-NaVβ1 experienced a highly significant reduction in spontaneous seizures compared with male *Scn1a*^+/−^ mice injected with AAV-EV (Fig. 4B; *t* = 3.43, *p* < 0.01). In contrast, there was no statistical difference in the frequency of tonic seizures in the female *Scn1a*^+/−^ mice treated with AAV-NaVβ1 compared with female *Scn1a*^+/−^ mice with AAV-EV (Fig. 4C; *t* = 0.75, *p* > 0.05).

Dravet mice, like patients with DS, are susceptible to heat-induced or febrile seizures. On PND 19 or 20, mice were tested for susceptibility to heat-induced seizures. Test mice were placed inside a customized testing chamber and the temperature was increased from 37°C to 42°C. The temperature at which a level 5 seizure (characterized by rearing and falling with forelimb clonus) occurred during the experiment was recorded. As expected in untreated DS mice, an increase in chamber temperature induced seizures in most of the *Scn1a*^+/−^ mice tested, whereas untreated WT mice showed no such effect (Fig. 4D). In the uninjected mice, there was no difference in seizure frequency between male and female mice. In the AAV-treated mice, there was no difference in the chamber temperature required to induce seizures in male (Fig. 4E) versus female (Fig. 4F) *Scn1a*^+/−^ mice injected with AAV-EV compared with those treated with AAV9-NaVβ1. The temperature ranges at which level 5 seizures occurred were also examined. Untreated *Scn1a*^+/−^ mice showed level 5 seizures between 40.5°C and 42°C, while AAV-EV- and AAV-NaVβ1-injected *Scn1a*^+/−^ mice had seizures between 38–42°C and 38.5–42°C, respectively. These observations indicate that AAV-NaVβ1 did not reduce the susceptibility of *Scn1a*^+/−^ mice to heat-induced seizures.

### Sex-dependent effects of AAV-NaVβ1 treatment on behavior

Because sex differences were observed in the mortality analysis and in seizure testing, male and female behaviors were analyzed separately. We focused on behavioral tasks that assessed autism-like behaviors such as motor activity, anxiety/innate fear, and learning and memory. Motor activity was examined in the open field test, the elevated plus maze test was used to examine anxiety/innate fear, and a passive-avoidance task was used to measure learning and memory.

**Figure 5.**
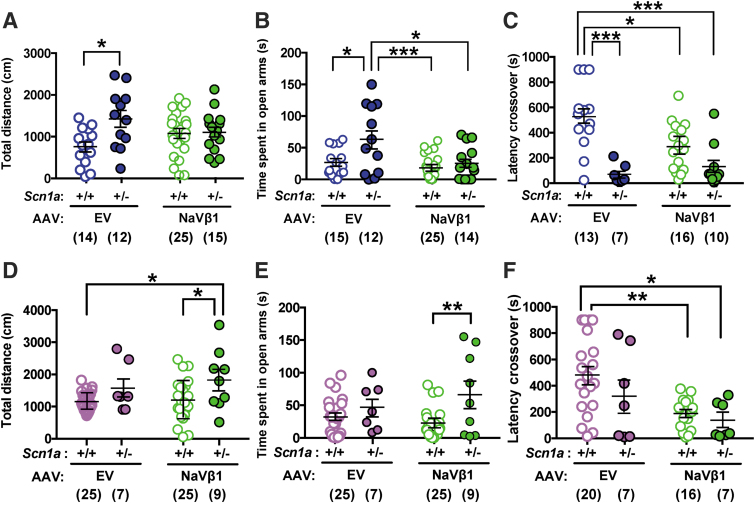
Results of behavioral testing in WT and *Scn1a*^+/−^ AAV-treated mice. **(A)** Results of the open field test comparing male WT and *Scn1a*^+/−^ mice treated with AAV-EV or AAV-NaVβ1. **(B)** Elevated plus maze results from male treated mice. **(C)** Results from male mice in the passive avoidance test. **(D)** Results of the open field test comparing total distance traveled in female WT and *Scn1a*^+/−^ mice treated with AAV-EV or AAV-NaVβ1. **(E)** Elevated plus maze results from treated female mice. **(F)** Results from the passive avoidance test in treated female mice. The data were analyzed with two-way analysis of variance and the Tukey *post hoc* test. *p < 0.05; **p < 0.01; ***p < 0.001, Tukey *post hoc* test.

In the open field test, male *Scn1a*^+/−^ mice treated with the control AAV-EV traveled farther than WT mice treated with AAV-EV; this difference was negated in *Scn1a*^+/−^ mice treated with the therapeutic AAV-NaVβ1 (Fig. 5A; treatment × genotype interaction, *F*_1,62_ = 5.37, *p* < 0.05; genotype main effect, *F*_1,62_ = 6.27, *p* < 0.05; at Tukey *post hoc* test, AAV-EV-treated WT vs. Scn1a^+/−^, *p* < 0.05). In the elevated plus maze, *Scn1a*^+/−^ mice injected with AAV-EV spent more time in the open arms compared with WT mice injected with the same vector. In contrast, WT and *Scn1a*^+/−^ treated with AAV-NaVβ1 did not differ in the time spent in the open arms, indicating correction of this endophenotype after treatment with AAV-NaVβ1 (Fig. 5B; treatment main effect, *F*_1,62_ = 3.66, *p* < 0.01; genotype main effect, *F*_1,62_ = 8.08, *p* < 0.01). Learning and memory were assessed using the passive avoidance test. Male *Scn1a*^+/−^ mice injected with AAV-EV took less time to enter into the dark compartment compared with WT littermates treated with AAV-EV (Fig. 5C; treatment × genotype interaction, *F*_1,42_ = 5.82, *p* < 0.05; genotype main effect *F*_1,42_ = 24.4, *p* < 0.0001), indicating impaired memory in *Scn1a*^+/−^ mice. However, treatment of *Scn1a*^+/−^ mice with AAV-NaVβ1 did not improve this impairment relative to *Scn1a*^+/−^ mice injected with AAV-EV (Fig. 5).

Analysis of behaviors in female mice indicated differences compared with results seen in males. In the open field test, female WT mice treated with AAV-EV did not differ from female *Scn1a*^+/−^ mice treated with the same vector, indicating no increased motor activity in the mutant mice compared with controls treated with the control vector (Fig. 5D). However, in the AAV-NaVβ1 treatment groups, *Scn1a*^+/−^ mice treated with AAV-NaVβ1 traveled farther than WT mice treated with either AAV-EV or AAV-NaVβ1 (Fig. 5D; genotype main effect, *F*_1,62_ = 9.32, *p* < 0.01). A similar outcome was observed in the elevated plus maze test where female *Scn1a*^+/−^ mice treated with AAV-EV did not differ from WT mice treated with the same vector (Fig. 5E), although in the AAV-NaVβ1-treated groups, *Scn1a*^+/−^ mice spent more time in the open arms compared with WTs treated with the same vector. (Fig. 5E; genotype main effect, *F*_1,60_ = 8.81, *p* < 0.01). The results of the passive avoidance test also showed no significant improvement in *Scn1a*^+/−^ mice injected with AAV-EV compared with WT injected with AAV-EV. Thus, unlike the male mice, the treated female mice showed a lack of phenotypic abnormalities in the baseline AAV-EV groups, and lack of a therapeutic effect in the AAV-NaVβ1-treated groups. We note that in a different line of Dravet mice, abnormal behaviors were also reported in untreated male, but not in female Dravet mice.^24^ In the passive avoidance test, female *Scn1a*^+/−^ mice treated with AAV-EV or with AAV-NaVβ1 both displayed reduced latency to crossover compared with WT mice injected with AAV-EV (Fig. 5F; treatment main effect, *F*_1,46_ = 1.90, *p* < 0.01). Taken together, these results indicate that several behavioral abnormalities observed in male mice were corrected by administration of AAV-NaVβ1; however, this was not seen in the treated female mice.

Finally, some older DS patients display abnormal motor function in the form of antecollis (disproportionate tonus of the neck muscles causing the head to droop forward) and a Parkinsonian gait.^25^ Although there are no previous reports of performance of *Scn1a* mutant mice on the rotarod test of motor coordination, rats with a homozygous mutation in *Scn1a* displayed impaired motor function on this test.^26^ However, we observed that compared with WT mice, untreated heterozygous *Scn1a*^+/−^ mice showed normal motor coordination and motor skill learning based on the rotarod test when assessed at 3 and 10 months (Supplementary Fig. S4).

## Discussion

We evaluated the ability of an AAV vector encoding the sodium channel NaVβ1 subunit to correct or mitigate the abnormal phenotype of the *Scn1a*^+/−^ mouse. Injection of AAV-NaVβ1 into the cerebral spinal fluid of neonatal mice caused overexpression of NaVβ1 and ameliorated several pathological endophenotypes. In contrast to the more robust prosurvival effect in treated female mice (see Sex differences in mortality in untreated *Scn1a*^+/−^ mice, and in response to treatment with AAV-NaVβ1 below), the results of the behavioral experiments showed correction in male *Scn1a*^+/−^ mice, whereas injected female *Scn1a*^+/−^ mice did not display behavioral correction. In adult males, motor hyperactivity and abnormal performance on the elevated plus maze were rectified after neonatal treatment with AAV-NaVβ1.

The promoter driving NaVβ1 expression was derived from a DNA sequence in the mouse *Gad-1* gene encoding Gad-67, an isoform of the GABA synthesizing enzymes expressed in GABAergic neurons throughout the brain. This customized promoter element displayed moderate GABA neuron selectivity in juvenile mice and lower selectivity in adult mice. Other GABA neuron-selective gene regulatory elements have been described, for example, the Dlx family of enhancers that participate in the development of GABAergic neurons in the telencephalon at early developmental stages.^27,28^ However, the Dlx elements might not be optimal for treating DS because they are reported to be operative only in forebrain regions,^27,28^ while NaV1.1 channels are found throughout the CNS, including in more caudal regions such as the cerebellum and brain stem.^13^

### Sex differences in mortality in untreated *Scn1a*^+/−^ mice, and in response to treatment with AAV-NaVβ1

Our results demonstrated that the untreated female *Scn1a*^+/−^ mice showed greater mortality than untreated male *Scn1a*^+/−^ mice. This finding was unexpected and, to our knowledge, sex differences in SUDEP in DS mutant mice have not previously been reported. The number of mice evaluated in the mortality analysis in the present study was relatively high (*n* = 28 males, *n* = 41 females), thereby revealing a robust difference (*p* < 0.01) in susceptibility of female *Scn1a*^+/−^ mice compared with males. Because NaV1.1 sodium channels are more highly expressed in GABA interneurons compared with excitatory neurons,^13,29^ and GABA-mediated inhibitory function is selectively compromised in Dravet mice,^15,16^ the higher susceptibility of female *Scn1a*^+/−^ mice might be linked to differential baseline inhibitory function between the sexes.

Evidence supporting this suggestion includes studies from WT rats and mice demonstrating lower inhibitory synaptic transmission in females. For example, Chudomel *et al.* reported higher inhibitory postsynaptic current frequencies, amplitudes, and charge transfer in WT male Sprague-Dawley rats compared with females,^30^ while WT female C57BL/6J mice showed lower GABAergic neuronal frequency and amplitude than male littermates.^31^ Additional evidence for innate differences in GABA-mediated inhibitory signaling between the sexes includes studies demonstrating lower GABA turnover, and lower GAD levels, thereby reducing GABA release in females compared with males.^32,33^ Another relevant parameter may be the effects of steroid hormones. Steroid hormones are potent modulators of GABA receptors, and GABA-mediated inhibition in the CNS and fluctuations of 17b-estrodiol, progesterone metabolites, and other female steroid hormones during the menstrual cycle may compromise inhibitory tone thereby inducing anxiety and increased seizure susceptibility.^34–36^

Based on these findings and our mortality results, we speculate that a lower level of endogenous GABA neurotransmission in females may contribute to comprise GABA interneuron function in *Scn1a*^+/−^ mice, thereby tipping the balance toward elevated incidence of SUDEP. The boost in survival observed in the female mice after treatment with AAV-NaVβ1 might be a reflection of the reported transient nature of GABAergic signaling deficit in *Scn1a*^+/−^ mice where inhibitory neurotransmission was profoundly impaired between postnatal days 12 and 35, but comparable with WT mice before and after those developmental time points.^37^ NaVβ1 overexpression during this critical period appeared to be sufficient to prevent SUDEP in some *Scn1a*^+/−^ mice.

Reports of the incidence of DS in males versus females in the human population have been mixed, with some indicating a predominance in females,^38^ while others reporting no difference in incidence between the sexes.^39^ In the epileptic condition known as severe myoclonic epilepsy in infancy, which has become synonymous with DS, a more severe form, termed “typical severe myoclonic epilepsy” was more common in females than males, while the less severe form, termed “borderline severe myoclonic epilepsy,” was more common in males.^40^ In terms of the incidence of SUDEP in the larger epilepsy patient population, the findings suggest a tendency for higher occurrence in females (15/25 female patients^41^; 15/26 females, including 10 patients treated with lamotrigine, 9 of whom were female^42^).

Thus, the reason for the discrepancy between our results demonstrating a clear elevation in susceptibility to SUDEP in female *Scn1a*^+/−^ mice, and the findings gleaned from SUDEP in DS patients, remains unknown. Nevertheless, one explanation could be the absence of anticonvulsant drug treatment in the present mouse study versus the unavoidability of anticonvulsant treatment in the clinical studies. Thus, despite the observation that most DS patients become refractory to antiepileptic medications in terms of suppressing seizures, anticonvulsants might protect against SUDEP in some patients. Of note in this context is the consensus that a first-line treatment for DS is clobazam,^9^ a benzodiazepine antiepileptic drug that potentiates GABA_A_ receptors and enhances GABAergic transmission.

### Therapeutic mechanisms of AAV-NaVβ1

NaVβ1 is a promiscuous and multifunctional protein that (1) potentiates channel activity of the pore-forming voltage-gated sodium channel α subunits,^43–46^ (2) functions as a chaperone of sodium channel α subunits from the intracellular pool to the neuronal membrane,^47^ (3) interacts with and modulates the activity of voltage-gated potassium channels,^48–50^ and (4) acts as a cell adhesion molecule to enhance neurite outgrowth.^51^ A population of NaVβ1 subunits are localized at the axon initial segment,^52^ as is NaV1.1.^13^ It is possible that overexpression of NaVβ1 increased NaV1.1 subunit expression on the neuronal plasma membrane from the large intracellular pool of subunits.^53^ Alternatively, we observed that the endogenous NaVβ1 protein was reduced in the untreated Scn1a^+/−^ mouse brain (Fig. 1C), and previous studies using Scn1b knockout mice showed that this line of mutant mice displayed spontaneous seizures and severe mortality.^54,55^ Thus, AAV-NaVβ1 administration in Scn1a^+/−^ Dravet mice likely rectified the deficit of NaVβ1, thereby contributing to the therapeutic efficacy.

Another possibility is that the rescue effects may have been mediated by direct sodium channel potentiation and/or modulation of potassium channels. NaVβ1 induces variable effects (either potentiation or inhibition) on several potassium channels, including Kv1.1, Kv1.2, Kv1.3, Kv1.6, Kv7 (Ref.^48^), and Kv4.2 (Ref.^49^). The Kv4.2 channel coimmunoprecipitates with NaVβ1 in the mouse brain, and in NaVβ1 knockout mice, action potentials in excitatory cortical pyramidal neurons are increased.^49^ This suggests that overexpression of NaVβ1 may suppress excitatory neuron firing, possibly by effects on Kv4.2. The promoter used in the present study showed expression of the NaVβ1 transgene in both GABA interneurons, where it would be expected to potentiate residual NaV1.1 sodium channels, and in non-GABA excitatory neurons where it may have dampened action potential firing via interaction with Kv4.2. These concomitant but reciprocal effects on sodium and potassium channels could have produced the therapeutic effect observed here in DS mice.

Our results demonstrate that a single treatment via AAV injection into the cerebral spinal fluid of neonatal mice produced therapeutic effects in juvenile and adult *Scn1a*^+/−^ mice. In addition to mutations in *SCN1A*, DS is also caused by mutations in the *SCN1B* gene coding for NaV1β.^54^ Thus, treatment with AAV-NaVβ1 could also be beneficial for this patient population. Finally, the long-term transgene expression and protracted therapeutic effects of AAV-mediated gene therapy reported in other studies, which in some cases lasted for years after a single injection,^56,57^ represent a major advantage over small-molecule-based anticonvulsant drugs that are typically given repeatedly and can be linked to loss of efficacy.

## Supplementary Material

Supplemental data

Supplemental data

Supplemental data

Supplemental data

Supplemental data
