# Supplementary material for: Sexually Divergent Mortality and Partial Phenotypic Rescue After Gene Therapy in a Mouse Model of Dravet Syndrome
Source: Hum Gene Ther. 2020 Mar 17;31(5-6):339–51. doi: 10.1089/hum.2019.225 (PMC7087406; doi:10.1089/hum.2019.225)
Supplement: Supplemental data [file Supp_Fig1.pdf]

Niibori et al. 2019
Supplemental figures and table

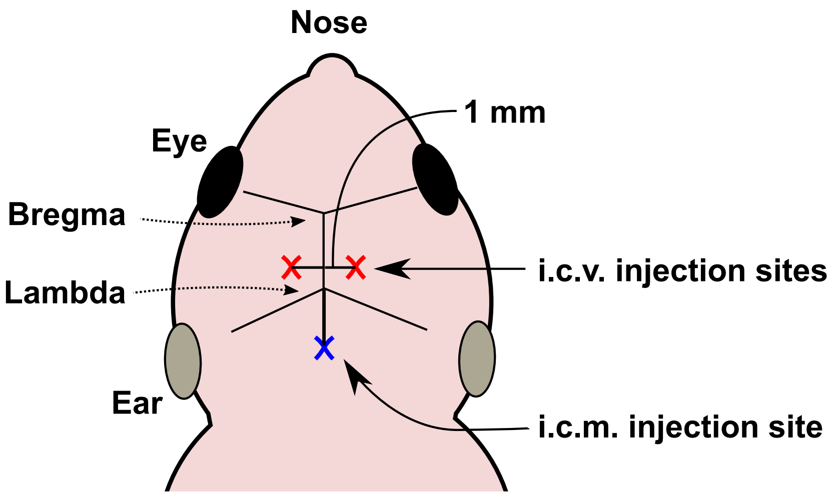

View from the top

FIGURE S1. Intra-CSF injections

Injection sites in postnatal day 2 mouse pups.
For intra-cerebroventricular (i.c.v.) injection, the injection needle was inserted 1mm anterior from the lambda and 1mm lateral from the midline through the skin and the skull. For intra-cistana magna (i.c.m.) injection, the needle was inserted at 2mm posterior from the lambda.
