# Supplementary material for: Sexually Divergent Mortality and Partial Phenotypic Rescue After Gene Therapy in a Mouse Model of Dravet Syndrome
Source: Hum Gene Ther. 2020 Mar 17;31(5-6):339–51. doi: 10.1089/hum.2019.225 (PMC7087406; doi:10.1089/hum.2019.225)
Supplement: Supplemental data [file Supp_Fig2.pdf]

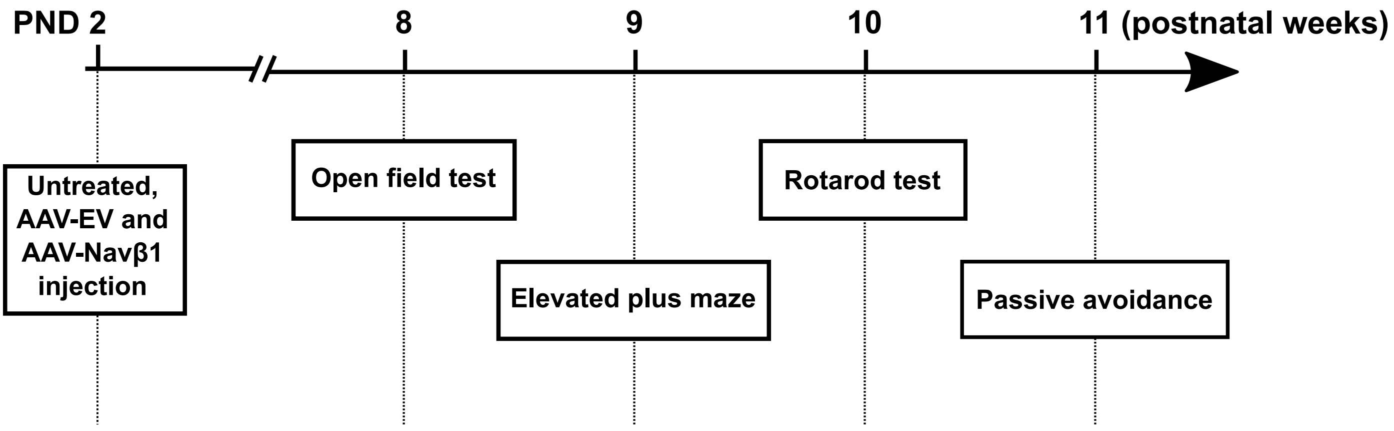

FIGURE S2. Temporal order of behavioral experiments

Mice with or without AAV treatment at postnatal day 2 were assessed for behavioral performance. In the 8th postnatal week, open field test was performed, followed by the elevated plus maze, rotarod test, and passive avoidance tests.
