# Supplementary material for: Sexually Divergent Mortality and Partial Phenotypic Rescue After Gene Therapy in a Mouse Model of Dravet Syndrome
Source: Hum Gene Ther. 2020 Mar 17;31(5-6):339–51. doi: 10.1089/hum.2019.225 (PMC7087406; doi:10.1089/hum.2019.225)
Supplement: Supplemental data [file Supp_Fig3.pdf]

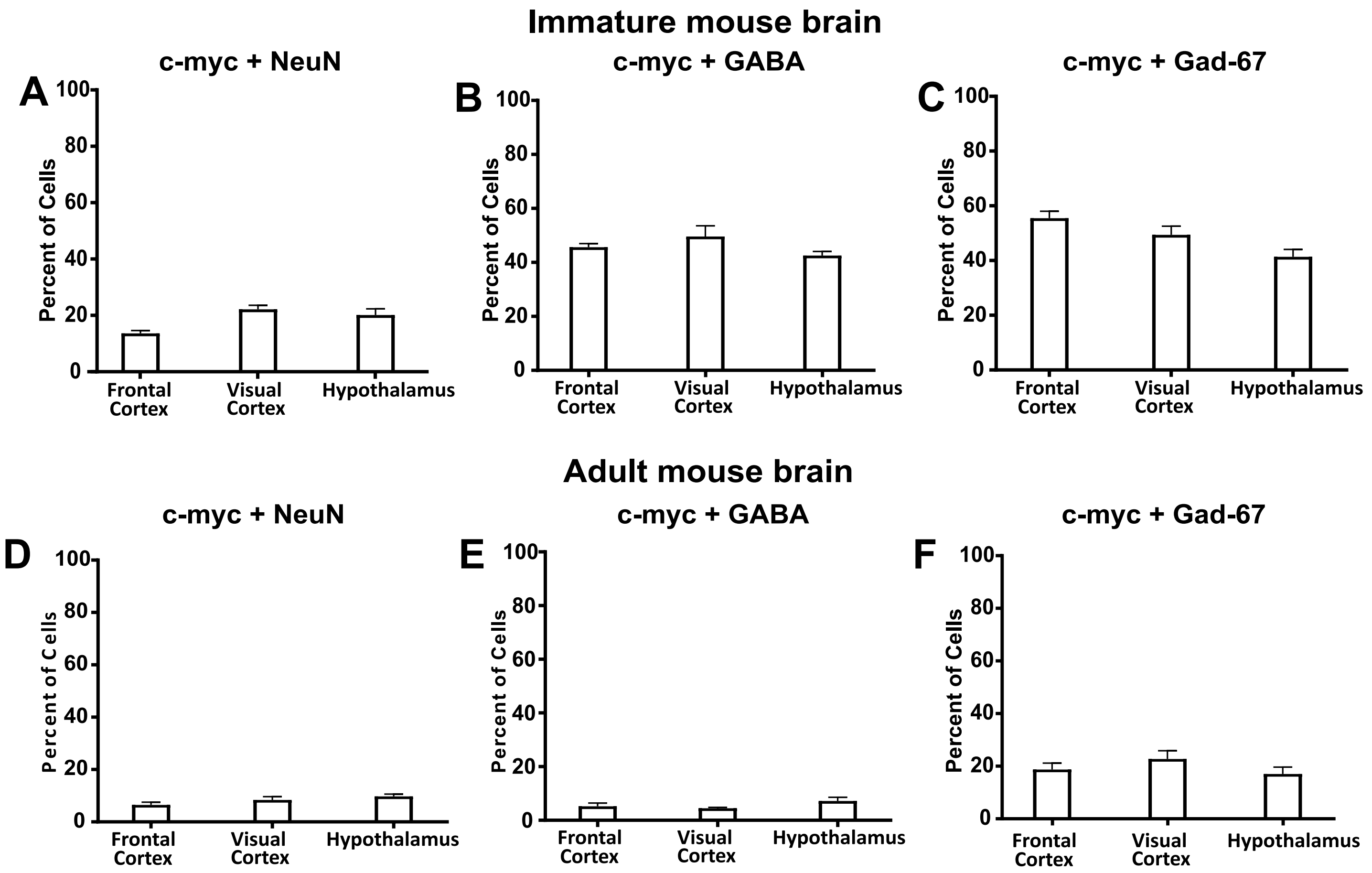

FIGURE S3. Cellular transduction coverage of NaVβ1 in immature and adult mouse brain.

The percentage of the total or GABAergic neurons that were transduced by NaVβ1-myc are presented as the cellular coverage. Panels A - F depict the cell coverage of the c-myc-tagged NaVβ1 transgene in the visual cortex, frontal cortex, and the hypothalamus. The bars represent the percentage of double positive cells in each of the NeuN, Gad-67, or GABA positive cell populations. Data in panels A - C summarize the results from the immature (P17-18) mice, while panels D - F correspond to the results obtained from adult mice. All values are means ± the standard error of the mean.
