# Supplementary material for: Sexually Divergent Mortality and Partial Phenotypic Rescue After Gene Therapy in a Mouse Model of Dravet Syndrome
Source: Hum Gene Ther. 2020 Mar 17;31(5-6):339–51. doi: 10.1089/hum.2019.225 (PMC7087406; doi:10.1089/hum.2019.225)
Supplement: Supplemental data [file Supp_Table1.pdf]

Summary of spontaneous seizure frequency in *Scn1a*$^{+/-}$ mice			
**Treatment**	**Gender**	**Seizures in mice that died prior PND 30**	**Seizures in mice that survived past PND 30**
**Untreated**	**Male**	**7.78 ± 3.37 (6)**	**0 (14)**
	**Female**	**6.90 ± 2.44 (8)**	**0 (3)**
**AAV-EV**	**Male**	**5.44 ± 1.23 (5)**	**0 (2)**
	**Female**	**5.5 ± 1.34 (8)**	**0 (9)**
**AAV-NaVβ1**	**Male**	**1.57 ± 0.20 (6)**	**0 (7)**
	**Female**	**4.11 ± 1.16 (6)**	**0 (3)**

TABLE S1. Spontaneous seizure frequency in *Scn1a*$^{+/-}$ mice

The numbers indicates the average number (± s.e.m.) of spontaneous seizures that occurred in *Scn1a*$^{+/-}$ mice at PND 30, or during the last 24 hours prior to death. The values shown in the brackets indicate the number of mice used in each experiment. The results were divided into two groups: *Scn1a*$^{+/-}$ mice that died during the video recording conducted between postnatal days 19-30, and *Scn1a*$^{+/-}$ mice that were still alive at PND 30.
