# Supplementary material for: Sexually Divergent Mortality and Partial Phenotypic Rescue After Gene Therapy in a Mouse Model of Dravet Syndrome
Source: Hum Gene Ther. 2020 Mar 17;31(5-6):339–51. doi: 10.1089/hum.2019.225 (PMC7087406; doi:10.1089/hum.2019.225)
Supplement: Supplemental data [file Supp_Fig4.pdf]

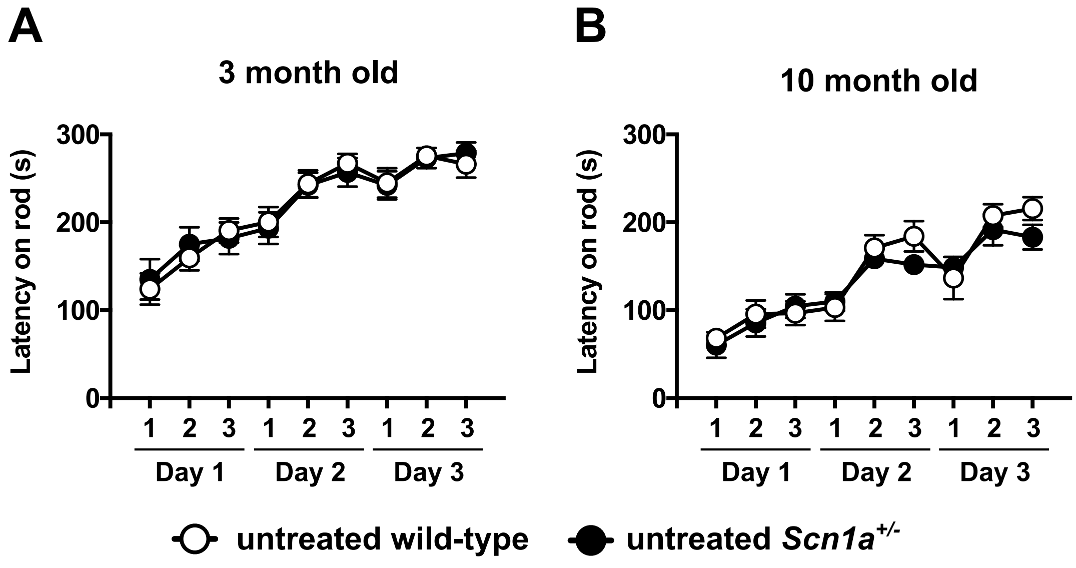

FIGURE S4. Rotarod test in untreated mice

Motor coordination and motor skill learning by accelerated rotarod test in wild-type and *Scn1a*$^{+/-}$ mice at 3 months old (a) and 10 months old (b). The performace at 1st trial of day 1 is compatible between wild-type and *Scn1a*$^{+/-}$ mice. The mice were increased in the latency on the rotating rod by repeated training trials (3 months old, trial main effect, $F_{8, 288} = 20.7$, $p < 0.0001$; 10 months old, trial main effect, $F_{8,135} = 20.12$, $p < 0.0001$). These results indicated that the motor performance of Scn1a +/- mice was similar to that of wild-type mice.
